# Effect of timing of umbilical cord clamping on anaemia at 8 and 12 months and later neurodevelopment in late pre-term and term infants; a facility-based, randomized-controlled trial in Nepal

**DOI:** 10.1186/s12887-016-0576-z

**Published:** 2016-03-10

**Authors:** Ashish KC, Mats Målqvist, Nisha Rana, Linda Jarawka Ranneberg, Ola Andersson

**Affiliations:** Department of Women’s and Children’s Health, International Maternal and Child Health, Uppsala University, Uppsala, SE-751 85 Sweden; United Nations Children’s Fund (UNICEF), Kathmandu, Nepal; Paropakar Maternity and Women’s Hospital, Kathmandu, Nepal; Department of Paediatrics, Hospital of Halland, Halmstad, Sweden

## Abstract

**Background:**

Delayed cord clamping at birth has shown to benefit neonates with increased placental transfusion leading to higher haemoglobin concentrations, additional iron stores and less anaemia later in infancy, higher red blood cell flow to vital organs and better cardiopulmonary adaptation. As iron deficiency in infants even without anaemia has been associated with impaired development, delayed cord clamping seems to benefit full term infants also in regions with a relatively low prevalence of iron deficiency anaemia. In Nepal, there is a high anaemia prevalence among children between 6 and 17 months (72–78 %). The objective of the proposed study is to evaluate the effects of delayed and early cord clamping on anaemia (and haemoglobin level) at 8 and 12 months, ferritin at 8 and 12 months, bilirubin at 2–3 days, admission to Neonatal Intensive Care Unit (NICU) or special care nursery, and development at 12 and 18–24 months of age.

**Methods/design:**

A randomized, controlled trial comparing delayed and early cord clamping will be implemented at Paropakar Maternity and Women’s Hospital in Kathmandu, Nepal. Pregnant woman of gestational age 34–41 weeks who deliver vaginally will be included in the study. The interventions will consist of delayed clamping of the umbilical cord (≥180 s after delivery) or early clamping of the umbilical cord (≤60 s). At 8 and 12 months of age, infant’s iron status and developmental milestones will be measured.

**Discussion:**

This trial is important to perform because, although strong indications for the beneficial effect of delayed cord clamping on anaemia at 8 to 12 months of age exist, it has not yet been evaluated by a randomized trial in this setting. The proposed study will analyse both outcome as well as safety effects. Additionally, the results may not only contribute to practice in Nepal, but also to the global community, in particular to other low-income countries with a high prevalence of iron deficiency anaemia.

**Trial registration:**

Clinical trial.gov NCT02222805. Registered August 19 2014.

## Background

At the time of birth, the infant is still attached to the placenta via the umbilical cord. The infant is usually separated from the placenta by clamping the cord with two clamps, and cutting between the clamps. This task takes place during the third stage of labour, which is the period of time from the birth of the infant to the delivery of the placenta [1].

Active management of the third stage of labour has been described in a recent World Health Organization (WHO) report as the “cornerstone” of obstetric and midwifery practice during the latter part of the 20th century [2]. Active management has involved the clinician intervening in the process of placental delivery through three interrelated practices: the administration of an uterotonic drug; early cord clamping and cutting; and controlled traction of the umbilical cord.

Early cord clamping has generally been advised to be completed within the first 30 s after birth, regardless of whether cord pulsation has ceased [3]. Due to evidence shown in the last decade, recent guidelines for active management of the third stage of labour no longer recommend immediate cord clamping [4], but changes in practice are still questioned [5] and policies in hospitals are rare [6].

Delayed clamping allows time for a transfer of the foetal blood in the placenta to the infant at the time of birth. This placental transfusion can provide the infant with an additional 40 % more blood volume [7]. The amount of blood transferred to the infant depends on when the cord is clamped and at what level the infant is held prior to clamping [8]. Neonatal benefits associated with this increased placental transfusion include higher haemoglobin concentrations, additional iron stores and less anaemia in early infancy and better cardiopulmonary adaptation [1, 9, 10].

Delayed cord clamping has, however, been linked to an increase in the incidence of jaundice which, in severe cases, could have longer-term effects on the health and development of the infant [1, 11].

Previous studies performed by the principal investigator in a high-income country have shown that delayed cord clamping, compared with early clamping, resulted in a reduced prevalence of neonatal anaemia [12]. Furthermore, delayed cord clamping improved iron status and reduced the prevalence of iron deficiency (ID) in infants at four months of age without demonstrable adverse effects [12–14]. As ID in infants even without anaemia has been associated with impaired development [15, 16], delayed cord clamping seems to benefit full term infants even in regions with a relatively low prevalence of ID anaemia [12].

The improved iron stores at four to six months after delayed cord clamping suggest that ID anaemia could be reduced at eight to twelve months of age, but this could not be shown in a later study by the principal investigators [17], possibly due to small sample size and low frequency of ID anaemia. However, delayed cord clamping was associated with improved fine motor function at 4 years of age [18]. Although ID anaemia is rare (3–9 %) in high-income countries [19], the negative impact on children’s health and development should not be underestimated. No randomized trial has evaluated the effect of delayed versus early cord clamping on infants after six months of age in a low-income country with high prevalence of ID and anaemia. As anaemia is associated with extensive health effects, such as stunting, fatigue and impaired neurodevelopment [20], reducing anaemia in infants is an urgent need globally. In an observational study from Peru, anaemia at eight months of age was evaluated in infants born before and after a hospital policy change from early to delayed cord clamping. The study resulted in a significant reduction of anaemia by 16 % (from 75 to 59 %) as well as a significantly higher level of haemoglobin among infants [21].

In Nepal, there is a high prevalence of anaemia among children aged 6–17 months (72–78 %) [22]. Approximately 50 % of all anaemia among pre-schoolers can be contributed to ID [23]. By performing the planned study in a country with high anaemia prevalence, we aim to evaluate any significant effects on haemoglobin levels and neurodevelopment outcomes after different timing of umbilical cord clamping in this high-risk population.

### Study objective

To evaluate the effects of delayed and early cord clamping on:

1. Anaemia (and haemoglobin level) at 8 and 12 months
2. Ferritin at 8 and 12 months
3. Bilirubin at 2–3 days
4. Admission to the NICU or special care nursery
5. Development at 12 and 18–24 months of age.

### Primary outcome

The primary outcome will be pre-specified infant haemoglobin at 8 month of age.

Anaemia will be defined as altitude corrected haemoglobin less than 110 g/L.

### Secondary outcomes

The secondary outcomes will be

- Haemoglobin at 12 months
- Ferritin at 8 and 12 months; definition iron deficiency as ferritin less than 12 μg/L
- Iron deficiency anaemia at 8 and 12 months, defined as both ferritin and haemoglobin below the respective cut offs
- Other outcomes will be hyperbilirubinemia at discharge, breast-feeding and morbidity during the first six months of life and psychomotor development at 12 months.

## Methods

### Study design and participants

This will be a randomized control trial in a hospital of Nepal with two parallel groups (1:1 ratio), delayed cord clamping (≥180 s) and early cord clamping (≤60 s). The study will be conducted in Paropakar Maternity and Women’s Hospital, a public funded tertiary centre for obstetric and gynaecological services in Kathmandu, Nepal.

In the hospital there are two separated delivery departments, high risk-labour room (LR) and low risk-Maternal and Neonatal Service Center (MNSC). At admission, according to the hospital protocol, the pregnant women are screened by an obstetrician who made the decision to which department the women will be transferred.

The hospital criteria for admission to MNSC are-uncomplicated pregnancies, no complication at the time of admission and healthy mothers (no clinical history of hypertension, infection, diabetes, chronic medical condition), expected vaginal delivery, gestational age between 34 and 41 weeks and singleton pregnancy.

**Fig. 1:**
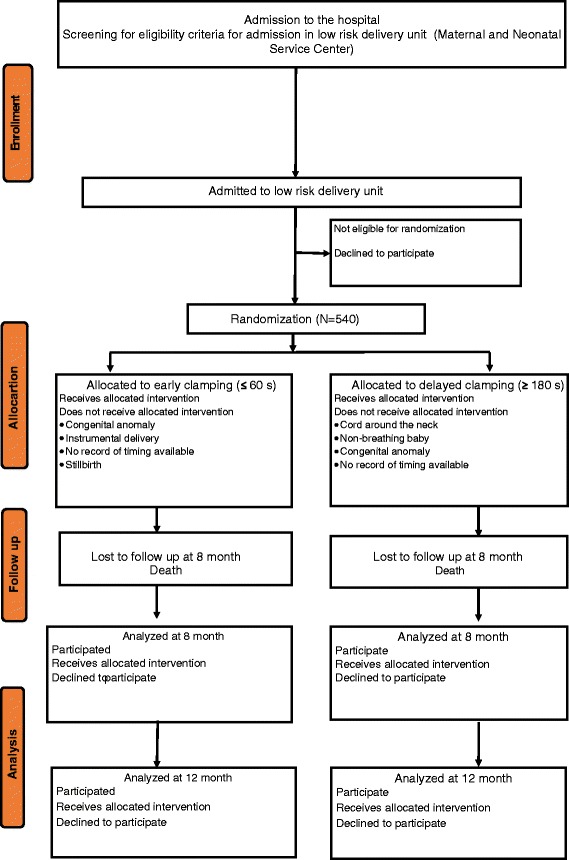
Trial profile (CONSORT flowchart)

Women will be eligible to participate in the study if they are assigned to MNSC. The exclusion criteria will be serious congenital malformation, syndromes, or the other congenital disease that could affect the outcome measures. Written consent will be obtained from the women who were eligible and willing to participate (Fig. 1).

### Randomization

The principal investigator will prepare a random list using random digit generator in Microsoft excel and will not have clinical involvement in the trial. Following this, he will prepare sequentially numbered, opaque envelopes and put in the colour cards with details of the allocated group and sealed the envelopes.

These will be kept at the research office and will be brought in the delivery unit before randomization.

Randomization will take place a few minutes before delivery when the nurse-midwife considered vaginal delivery is imminent. To allocate the women into treatment group, the surveillance officer (SO) will open the next consecutively numbered envelope and inform the nurse-midwife of the assigned treatment. The used colour card and envelope will be discarded.

### Surveillance protocol

Before the study period, early clamping was predominately practiced at the hospital. During the study period, surveillance officers (SO) are stationed 24 h a day at three stations, the reception, the delivery ward, and the postnatal wards. SO are all trained nurse-midwives.

As the pregnant woman arrives at the hospital, she will receive written information about the study in the reception. A SO will then approach the woman and ask for her consent to participate in the study.

If the woman agrees, she will sign the consent form and the SO stationed at the reception will register the information needed according to protocol. The woman’s labour will be managed by following the hospital’s ordinary routine until she is transferred to the delivery ward, where the randomisation takes place. The SO stationed at the delivery ward will pair the woman with a sealed, numbered, opaque envelope containing the treatment allocation and show this to the nurse-midwife conducting the delivery.

### Intervention

When delivery is imminent (expected within 10 min), the nurse-midwife will open the sealed, numbered, opaque envelope to reveal the treatment allocation. The interventions consist of delayed clamping of the umbilical cord (≥180 s after delivery) or early clamping of the umbilical cord (≤60 s). The SO will measure the time from complete delivery of the baby until the first clamp is placed on the umbilical cord with a stopwatch. All other aspects of obstetric care will be managed according to standard practice at the hospital. Should the infant need resuscitation, the umbilical cord will be clamped and cut and the infant will be carried to the resuscitation table for further handling. In both groups, oxytocin will be given to the mother after the umbilical cord is clamped. All staff in the delivery unit will be trained in the study procedures before the trial is started.

### Follow-up

After delivery, the babies will be cared for according to standard clinical routines, and early breast-feeding will be encouraged. As part of the study, the nurse-midwife will assess the infant at 1 and 6 h, to see whether the baby has been breastfed Infants will stay at the postnatal ward with their mothers for two or three days after delivery, except for those well babies whose mothers prefer to leave the hospital earlier and infants who require admission to the neonatal unit. The SO stationed at the postnatal ward will perform transcutaneous bilirubin measurements when child and mother are discharged from the hospital, as well as 24 and 48 h after delivery if possible. The following information will be collected from maternal healthcare records: background information of the mother’s age and parity, babies’ weight, gestational age and Apgar score.

Monthly, up to 12 months of age, a SO will call the family and ask questions regarding infections, breast-feeding and immunizations of the infant.

At 8 and 12 months of age, infants will be scheduled for a follow-up visit including blood sampling (haemoglobin and ferritin). Venous blood sampling will be performed. Mother’s will be interviewed about the infant’s feeding practice.

At 12 months of age, the Ages & Stages Questionnaire will be used to record infants’ neurodevelopment. Parents will be assisted in answering the Ages & Stages Questionnaire.

If funding is obtained, the Bayley Scales of Infant and Toddler Development, third edition will be used to examine children at 18–24 months of age.

### Blinding

The study design precluded either the mother giving birth or the nurse-midwife performing the intervention being blinded. Physicians performing neonatal examinations, staff members responsible for collection of blood samples and background data, and laboratory staff performing analyses of blood samples will be blinded to each infant’s allocation group.

### Sample size

The sample size for the primary outcome at eight months was estimated in order to find a difference of 15 % (70 versus 55 %) in the prevalence of anaemia between the two randomization groups with a power of 80 % and a type I error rate of .05. Using Fisher’s exact-test to analyse outcome data, a group size of 176 would be needed. Taking into account an attrition rate of 35 % we calculated that 270 newborns should be included in each group, i.e. a total of 540.

### Timeline

The study will begin on October 2, 2014 with the expected enrolment of the 540 participants within 45 to 60 days of the project start date.

### Data management

The SO will fill up and assess data records. The two research managers will verify all record forms with the primary source of data. A data entry officer will re-check them for discrepancies before entering the data in computers. The quality control team from Uppsala University will provide oversight to ensure quality of data collection and to avoid data loss. A protocol for data tracking will be followed. All cases will be analysed on an intention to treat basis.

The Census and Survey Processing System (CSPro), a public domain software package developed and supported by the U.S. Census Bureau and ICF Macro, will be used for quality data management. CSPro is interfaced with SPSS (originally, Statistical Package for the Social Sciences, 20), which will be used for statistical analysis, data management (case selection, file reshaping, creating derived data), and data documentation. Hard copies of records will be stored in a filing system in a secure room. Data will be checked for accuracy, consistency, and completeness in both CSPro and SPSS. An analysis plan will be developed in accordance with the reporting guidelines. A profile and comparison of key variables between groups at baseline will be presented.

### Ethical considerations

All research involving newborn infants and small children needs careful ethical consideration, mainly since the subjects themselves cannot agree to whether they want to participate in the study or not. In particular, research that is not immediately beneficial for the patients needs special ethical consideration. The included infants are healthy full-term infants undergoing umbilical cord clamping, which is a standard procedure after birth. The possible benefit of the intervention (delayed cord clamping) for increasing iron stores and preventing infant anaemia is estimated to be higher than the risk of probable adverse effects, such as hyperbilirubinemia. Ethical approval has been sought and obtained from Nepal Health Research Council (Reg no. 76/2014) on 5 June 2014. The trial has been registered at clinicaltrial.gov with the registration number NCT 02222805 on August 19, 2014. Written informed parental consent will be obtained before the intervention is given, and parents can withdraw from the study at any time without any need for explanation.

## Discussion

Reducing ID among infants is important, as it is associated with impaired neurodevelopment [16]. With a high global prevalence of infant anaemia, delayed cord clamping has the potential to reduce infant anaemia and thereby improve infants’ and children’s health and development. In crude numbers, a reduction by 10 % would mean an annual reduction of 60,000 infants with anaemia in Nepal. This trial is important to perform because, although strong indications for the beneficial effect of delayed cord clamping on anaemia at 8 to 12 months of age exist, it has not been evaluated by a randomized trial in a low-income setting with a high prevalence of ID and anaemia. By completion of the proposed study, both outcome as well as safety effects will be analysed. Additionally, the results may not only contribute to the practice in Nepal, but also to the global community, in particular to other low-income countries with a high prevalence of ID anaemia.

## Abbreviations

UNICEF: United Nation’s Children’s Fund
NICU: Neonatal Intensive Care Unit
NCT: National Institute of Health Clinical Trial
WHO: World Health Organization
ID: iron deficiency
SO: surveillance officers
CSPro: Census and Survey Processing System
SPSS: Statistical Package for the Social Sciences
